# iTRAQ Quantitative Proteomic Analysis of Different Expressed Proteins and Signal Pathways in Bakuchiol-Induced Hepatotoxicity

**DOI:** 10.1155/2022/2928240

**Published:** 2022-09-19

**Authors:** Shu-Yan Gao, Deng-Qiu Xu, Abudumijiti Abulizi, Youlidouzi Maimaiti, Silafu Aibai, Zhen-Zhou Jiang, Lu-Yong Zhang, Zhi-Jian Li

**Affiliations:** ^1^Uyghur Medicines Hospital of Xinjiang Uyghur Autonomous Region, Urumqi 830049, China; ^2^College of Pharmacy, Xinjiang Medical University, Xinjiang Uyghur Autonomous Region, Urumqi 830054, China; ^3^Xinjiang Institute of Traditional Uyghur Medicine, Urumqi 830049, China; ^4^Jiangsu Key Laboratory of Drug Screening, China Pharmaceutical University, Nanjing 210009, China; ^5^State Key Laboratory of Natural and Biomimetic Drugs, Department of Pharmacology, School of Basic Medical Sciences, Peking University, Beijing 100191, China; ^6^Center for Drug Screening and Pharmacodynamics Evaluation, School of Pharmacy, Guangdong Pharmaceutical University, Guangzhou 510006, China

## Abstract

Bakuchiol (BAK) is an abundant natural compound. BAK has been reported to have several biological activities such as anticancer, antiaging, anti-inflammatory, and prevention of bone loss. However, it causes hepatotoxicity, the mechanism of which is not known. In this study, we explored the mechanism of BAK hepatotoxicity by treating rats with 52.5 mg/kg and 262.5 mg/kg of BAK, administered continuously for 6 weeks. We examined the liver pathology and biochemical composition of bile to determine toxicity. Mechanisms of BAK hepatotoxicity were analyzed based on relative and absolute quantification (iTRAQ) protein equivalent signatures and validated in vitro using LO2 cells. iTRAQ analysis revealed 281 differentially expressed proteins (DEPs) in liver tissue of the BAK-treated group, of which 215 were upregulated, and 66 were downregulated. GO and KEGG enrichment analysis revealed that bile secretion, lipid metabolism, and cytochrome P450 signaling pathways were enriched in DEPs. Among them, peroxisome proliferator-activated receptor *α* (PPAR*α*), farnesoid *X* receptor (FXR), and cholesterol 7*α*-hydroxylase (CYP7a1) were closely associated with the development and progression of BAK-induced hepatic metabolic dysfunction and abnormal bile metabolism. This study shows that BAK can induce hepatotoxicity through multiple signaling pathways.

## 1. Introduction

**Figure 1:**
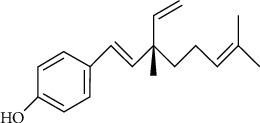
The structural formula of bakuchiol (BAK).

BAK (Figure 1) is a major monoterpenoid compound isolated from the fruit of Fructus Psoraleae (FP). It has been proved to have an antibacterial, anti-inflammatory, antioxidant, estrogen-like effect and an anticoagulant effect of neutralizing snake venom [1]. It can be used as a protective drug to prevent diabetes, bone loss, benign prostatic hyperplasia [2], and neurological diseases [3]. FP has xerogenic and strong hepatotoxicity. In recent years, with the in-depth study of FP and more and more clinical reports of adverse reactions, the FP main active components have also aroused widespread concern. There was news that 158 patients had adverse reactions of severe liver injury [4] due to taking the traditional Chinese medicine Zhuang-Gu-Guan-Jie-Wan containing FP, as reported by the 16th issue of the adverse drug reaction (ADR) information announcement and the China Food and Drug Administration (CFDA). There were 11 patients with liver injury after taking Baishi tablets containing FP from 1978 to 2005 in China [5]. In clinical reports, FP can cause abnormalities of liver biochemical indicators, such as alanine aminotransferase (ALT), total bilirubin (TBIL), and direct bilirubin (DBIL). Intrahepatic cholestasis can be seen in some patients by liver puncture pathological examination. BAK is classified as a toxic compound that may cause FP hepatotoxicity.

Drug-induced liver injury (DILI) is one of the major problems in drug development and chemical safety, with abnormal bile metabolism accounting for almost half of all DILI cases, showing high morbidity and mortality. The pathophysiology mechanism is still unclear, but drug-induced liver injury in the form of cholestasis can be caused by inhibition of the hepatobiliary transport system mediated by drugs or metabolites. The previous research results of the research group showed that BAK can induce cholestatic hepatotoxicity through its influence on liver lipid metabolism. High-dose BAK can cause an increase in glutamic-oxalacetic transaminase (AST) and a decrease in ALT, lactate dehydrogenase (LDH), cholesterol (TC), and triglyceride (TG), which has obvious harmful effects on liver lipid metabolism [6]. It may be related to the disorder of 3-hydroxy-3-methylglutaryl reductase (HMG-CoA) lipid metabolism in the RohA pathway and PPAR*α*-induced liver *X* receptor *α* (LXR*α*) expression. However, due to the limitations of research methods, only a single or several differentially expressed proteins in the liver are concerned. Systematic changes in liver at the molecular level during injury have not been explored.

Quantitative proteomics is a relatively new way to study the etiology of a disease. This technology can be used to identify and quantify more unique proteins in biological samples [7]. In the study of FP's liver toxicity in rats, we used this method to systematically evaluate the DEPs in the liver induced by ethanol extract of psoralen. The results showed that FXR, PPAR, and CYP7a1 are closely related to the occurrence and development of liver metabolic dysfunction and cholestasis caused by ethanol extract of FP (EEFP). FP is rich in a variety of chemical components and has the characteristics of multicomponent and multitarget. BAK is the main active ingredient in FP. To elucidate the mechanism of the toxic mechanism of BAK can provide a reference for the subsequent molecular biological studies on the mechanism of hepatotoxicity of FP and BAK and clinical safe medication. In the current study, iTRAQ quantitative proteomics was used to analyze liver tissue samples from the BAK-treated group and compare them to control group liver tissue samples. Biliary biochemistry, specific damage indicators, and pathological changes in target tissue (target cell) are combined to screen for differentially expressed genes or proteins to provide molecular signals associated with toxicity and regulatory networks of related genes and proteins. Such analysis can provide a lot of information on the mechanism of BAK liver toxicity.

## 2. Materials and Methods

### 2.1. Drugs, Chemicals, and Antibodies

BAK content ≥98% was obtained from Jing Zhu Bio-Technology Co., Ltd., Nanjing, China. Other reagents were of high-analytical grade and commercially available. The following antibodies were used in this study: SLC10A1 (NTCP, bs-1958R), NR1H4 (FXR, bs-22519R), PPAR*α* (bs-3614R), NR0B2 (SHP, bs-4311R), ABCB11 (bs-4311R), and *β*-actin (bs-0061R), which were purchased from Bioss Inc. (Beijing, China), and CYP7a1 (D161909), which was purchased from Sangon Biotech Co., Ltd. (Shanghai, China).

### 2.2. Animals and Treatment

Objects of the study are SD rats (*n* = 30, half male and female, 130–150 g, specific pathogen-free), and the protocols for the experiments were approved by the Xinjiang Institute of Traditional Uygur Medicine Ethics Committee on Animal Experimentation (production certificate no. SCXK2016-0003, Xinjiang, China) and were conducted in accordance with China's National Animal Health and Medical Research Council regulations for animal care and use for scientific purposes. The rats were raised in the drug safety evaluation center of SPF animal room of Xinjiang Uygur Medical Research Institute. The environmental conditions (12 h light/dark cycle, temperature 24°C, and humidity 40–70%) were controlled to provide standard food and drinking water for experimental animals. The experiment rats were acclimatized for 3 days and subsequently divided into 3 groups (*n* = 10, half male and female): group 1 included rats orally dosed with olive oil (control group), and groups 2 and 3 included rats orally dosed with different doses of BAK (52.5 or 262.5 mg/kg body weight, BAK-L and BAK-H, resp.) in an experiment that lasted for 6 weeks. The rats were periodically observed for clinical signs of toxicity, mortality, morbidity, body weight changes, and feed consumption. Six weeks after treatment, rats were subjected to a bile collection after fasting for 12 hr.

### 2.3. Bile Output and Component Analysis

The rats were anesthetized with pentobarbital, and thin polyethylene tubing was inserted into the common bile duct [8]. After a 2 min equilibration, bile was collected for 2 hr to determine the total bile output, flow rate, and bile constituents. Total bile acid (TBA), DBIL, and TBIL were determined using a HITACHI7080 Automatic Clinical Analyzer (Tokyo, Japan), and all kits were purchased from Mindray Biomedical Electronics Co., Ltd. (Shenzhen, China).

### 2.4. Histological Analysis

The liver tissue specimens were fixed in formaldehyde buffer solution (10%) for 24 hours. After staining with hematoxylin and eosin (H&E), the slices are checked blindly by a senior pathologist.

### 2.5. iTRAQ Quantitative Proteomics Analysis

#### 2.5.1. Protein Extraction, Digestion, and iTRAQ Labeling

The iTRAQ study was conducted on the control group and the high-dose treatment group. Liver tissue (50 mg) was taken and placed in 1 ml SDT buffer (containing 4% SDS, 100 mM DTT, and 100 mM Tris-HCl, pH 7.6) and homogenized (24 × 2, 6.0 M/S, 60 S, twice). The liver homogenate was ultrasonicated at 80 W for 10 times and centrifuged at 14,000*g* and 4°C for 15 min. The supernatant was filtered with a 0.22 *μ*m filter membrane, and the filtrate was determined by a BCA kit (Beyotime Biotech, Nanjing, China). After protein quantification, 30 *μ*g of protein solution was alkylated with 100 mM iodoacetamide in UA buffer at room temperature (RT) for 30 min. The alkylated protein solution was added to 200 *μ*L UA buffer, mixed well, then transferred into a 30 kD ultrafiltration centrifuge tube, and centrifuged at 14000*g* for 15 min, and the filtrate was discarded (repeat this step once). The protein samples were washed twice with 100 *μ*L UA buffer and 0.5 M triethylammonium bicarbonate (pH 8.5) solution buffer. The obtained samples were digested with 40 *μ*L trypsin (4 *μ*g sequenced-grade modified trypsin dissolved in 40 *μ*L solution buffer) at 37°C for 18 h. Mixed peptide fragments were collected and centrifuged at 14000*g* for 15 min for peptide quantification (Nano Drop 2000). 100 *μ*g of peptides was taken from each sample and labeled according to the instructions of the iTRAQ Regent 8-plex Multiplex Kit (AB Sciex, Framingham, MA, USA). The fractions were lyophilized by a rotary vacuum concentrator and desalted by a C18 cartridge (66872-U, Sigma, St. Louis, MO, USA).

#### 2.5.2. Strong Cation-Exchange Fractionation and LC-MS/MS Analysis

Each group labeled peptide was mixed and loaded onto the analytical column (Thermo Scientific, Acclaim PepMap RSLC 50 um × 15 cm nanoviper P/N164943) by the Agilent 1260 Infinity II HPLC system (Agilent, Santa Clara, CA, USA). The iTRAQ-labeled peptides were separated by a linear gradient formed by mobile phase A (10 mM HCOONH4, 5% ACN, pH 10.0) and mobile phase B (10 mM HCOONH4, 85% ACN, pH 10.0). The elution was accomplished with a solvent flow rate of 1 *μ*L/min, using a gradient program as follows: 0–25 min, 5% B; 25–30 min, 20% B; 30–65 min, 40% B; 65–70 min, 60% B; 70–85 min, 100% B; and 60–75 min, 100% A. During the elution process, we monitored the absorbance value of 214 nm, collected the eluted components every 1 min, and received 36 eluted components.

Peptide separation of the individual SCX fractions was performed on a Proxeon Easy-nLC 1000 system (Thermo Scientific, San Jose, CA, USA). Peptide mixtures were first loaded onto precolumn (20 mm × 100 *μ*m, 5 *μ*m C18; Thermo Scientific, San Jose, CA, USA) and then separated on analytical EASY columns (75 *μ*m × 100 mm, 3 *μ*m C18; Thermo Scientific, San Jose, CA, USA). Solvents used 0.1% formic acid (mobile phase A) and 0.1% formic acid in 80% acetonitrile (mobile phase B). Tryptic peptides were eluted through a multistep gradient from 0% to 38% buffer B with a linear gradient at 0–50 min; 38% to 100% buffer B with a linear gradient at 50–55 min; 100% buffer B at a flow rate of 0.3 *μ*L/min at 55–60 min.

The eluted tryptic peptides were directly interfaced with a Q Exactive Mass Spectrometer (Thermo Fisher Scientific, Waltham, MA, USA). Data were acquired in positive ion mode with a selected mass range of 350–1800 mass/charge (m/*z*). Q Exactive survey scans were acquired at a resolution of 70,000 (m/*z* 200), the resolution for higher-energy collisional dissociation (HCD) spectra was 17,500 (m/*z* 200), and the maximum ion injection time was fixed at 20 and 60 ms. The dynamic exclusion duration was 40 s. The normalized collision energy was 30 eV, and the underfill ratio was defined as 0.1% [9]. Each fractionation was performed in triplicate LC-MS/MS runs.

#### 2.5.3. Data Analysis

Raw mass spectrometry data were identified and quantified using the software Mascot 2.2 (Matrix Science, London, UK) and the UniProt website (https://www.uniprot.org). Proteins with quantification *p* value < 0.05 and with fold change ≥1.2 or ≤0.833 (the average fold change of three repeat experiments) were considered as DEPs. The gene ontologies (biological processes and molecular functions) of all IDs were searched against the Gene Ontology database using Blast2GO-Functional Annotation and Genomics (https://www.geneontology.org) as well as KEGG Mapping-GenomeNet (https://www.genome.jp/kegg/). Hierarchical clustering was performed using Cluster 3.0/Java TreeView, and heatmaps were generated. Pathway analyses for protein-protein interactions and upstream regulations of differentially expressed candidates were performed using Cytoscape 3.2.1 software.

### 2.6. Cell Lines, Culture Conditions, and Viability Assay

L02 cell is widely used as an in vitro model of nonmalignant liver. L02 was purchased from the Cell Bank of the typical Culture Preservation Committee of the Chinese Academy of Sciences (Shanghai, China). Both cells were cultured in Dulbecco's Modified Essential Medium (DMEM, Gibco, USA) supplemented with 10% fetal bovine serum (Gibco, USA), 100 U/mL penicillin, and 100 U/mL streptomycin in a humidified environment at 37°C with 5% CO2, and the cell growth was observed regularly. It was digested with 0.25% trypsin every 2 days and subcultured. For the cell viability assay, cells were seeded in 96-well plates at a density of 3000 cells/well. After treatment with BAK (1–100 *μ*M) for 48 hr, cell viability was measured using the 3-[4,5-dimethylthiazol-2-yl]-2,5-diphenyltetrazolium bromide (MTT) assay.

### 2.7. Quantitative Real-Time PCR Analysis

Total RNA of L02 cells was extracted using TRIzol (Invitrogen Life Technologies, Carlsbad, CA) and the RNApure Tissue&Cell Kit (CWBIO, Taizhou, China) according to the manufacturer's instructions. Total RNA was subjected to cDNA synthesis using PrimeScript RT Master Mix (Takara Biotechnology, Dalian, China). PCR was performed in a volume of 20 *μ*L containing 10 *μ*L of SYBRGreen Supermix (Vazyme Biotech, Nanjing, China), 1 *μ*L of cDNA, 7 *μ*L of RNase/DNase-free water, and 500 nM of each primer. Primers for genes CYP7a1, bile-salt export pump (BSEP), small heterodimer partner (SHP), FXR, and *β*-actin were synthesized (Sunshine Biotechnology, Nanjing, China), and the primer pairs were listed in Table 1. Gene expression was evaluated by the ΔΔCT method, using *β*-actin (for human genes) as reference genes.

### 2.8. Differential Protein Expression by Western Blot

Protein extraction of cells was performed using a whole Protein Extraction Kit (KeyGen Biotech, Nanjing, China). The protein concentration in the extract was measured using a bis-octanoic acid (BCA) Protein Assay Kit (Beyotime Biotechnology, Nanjing, China). Equal amounts of total protein (6–10 *μ*L) were separated by electrophoresis on each channel with 8% or 12% SDS polyacrylamide gels. After electrophoresis, the gel was transferred to a nitrocellulose membrane (Millipore, Billerica, MA, USA) and then blocked with 5% skim milk for 1 hr at room temperature to block nonspecific sites. The skim milk was washed and removed in TBST (containing 0.1% Tween 20 in TBS buffer) to avoid contamination of the antibody. Then, the membranes were incubated overnight at 4°C in a solution containing 0.1% Tween 20, 5% Bovine Serum Albumin (BSA), and a dilution of the primary antibodies (1 : 500–1000). After four washes in TBST, the membranes were incubated with horseradish peroxidase- (HRP-) conjugated secondary antibodies for enhanced chemiluminescence (ECL) detection using a Super Signal Substrate kit (Pierce Chemicals, Rockford, IL, USA). Signals were detected using a Tanon 4200 chemiluminescence imaging system (Shanghai, China). *β*-Actin, PPAR*α*, CYP7a1, SHP, FXR,Na/taurocholate cotransporting polypeptide (NTCP), and p-acetyl-CoA carboxylase (ACC) primary antibody could cross-react with the human proteins and were used in samples from the human L02 cell line. Specimens were assessed for *β*-actin content as an internal control.

### 2.9. Statistical Analyses

Results were analyzed using the one-way analysis of variance (ANOVA) or Dunnett's *t*-test (SPSS software package 25.0). All data represent at least 3 independent experiments and are shown as the mean ± SEM. The values with *p* < 0.05 were considered statistically significant.

## 3. Results

### 3.1. BAK Affects Biliary Secretion of Bile Acids (BAs) In Vivo

**Figure 2:**
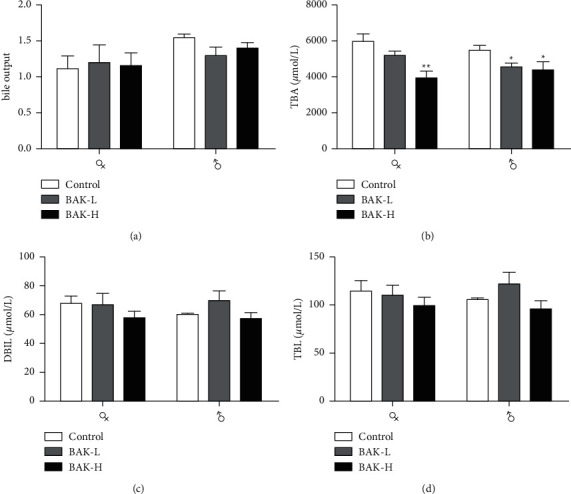
Changes in bile output and bile composition. (a) Bile output. (b) Variation in biliary TBA output. (c) Variation in biliary DBIL output. (d) Variation in biliary TBIL output. The values represent as mean ± SEM (*n* = 5). ^*∗*^*P* < 0.05 and ^*∗∗*^*P* < 0.01 versus the control.

The biliary secretion and bile composition were further analyzed for the changes relevant to the injury of the liver. The decrease of TBIL and DBIL output in bile was consistent with elevated biliary secretion in BAK-treated groups (Figures 2(a), 2(c), and 2(d)). TBIL and DBIL output in the male BAK-L group showed an increasing tendency. Interestingly, the output of TBA significantly decreased under the action of BAK, especially in the female of the BAK-H group, and it was dose-dependent (Figure 2(b)). This may have an important relationship with BAK's influence on cholesterol metabolism and bile metabolism.

### 3.2. Histopathological Changes

**Figure 3:**
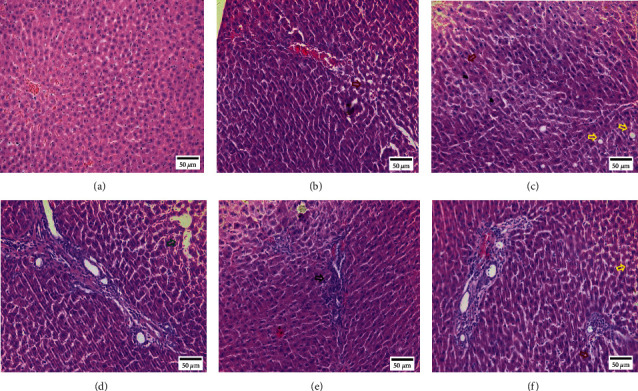
Effect of 6 weeks of BAK exposure on rats' hepatotoxicity (H&E staining). Cholestasis is shown by the red arrow; steatosis is shown by the yellow arrow; bile duct proliferation is shown by the green arrow; inflammatory cell infiltration is shown by the black arrow. (a) Control, (b) BAK-L, (c) BAK-L, (d) BAK-H, (e) BAK-H, and (f) BAK-H.

Compared with the control group, the liver tissue of the BAK group had pathological changes. Mainly manifested as different degrees of hepatocyte steatosis, interstitial inflammatory cell infiltration, and cholestasis lesions, mild hepatic steatosis and cholestasis were observed in the BAK-L group. In the BAK-H group, fatty degeneration of hepatocytes, cholestasis, inflammatory infiltration of hepatocytes, and bile duct hyperplasia were observed, as shown in Figure 3.

### 3.3. Identification of Differentially Expressed Proteins in Liver Tissue of Control versus BAK-H Groups

From the iTRAQ-based quantitative proteomic analysis of liver tissues from the control and BAK-H groups, a total of 281 proteins were determined as DEPs, of which 215 DEPs were upregulated and 66 DEPs downregulated. Proteins with quantification *P* value < 0.05 and with fold change ≥1.2 or ≤0.833 (the average fold change of three repeat experiments) were considered as DEPs.

### 3.4. GO and KEGG Pathway Enrichment Analysis

**Figure 4:**
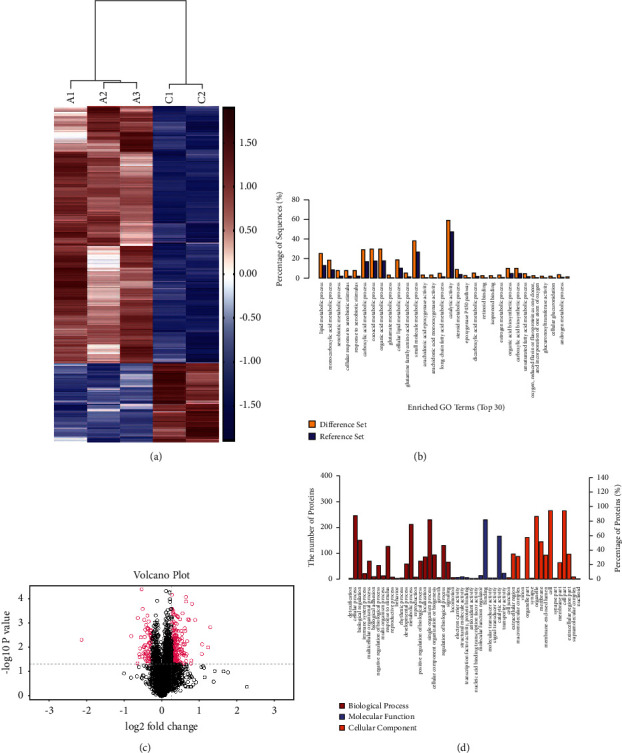
Proteomics analysis of EEFP-treated rats. (a) Heatmap of the significantly altered proteins in the control and BAK treatment groups. Red indicates upregulation, and blue represents downregulation in protein expression. (b) GO analysis results (difference set: control group; reference set: BAK group). (c) Volcano plots of the proteins quantified during iTRAQ analysis comparisons. Each point represents the difference in expression (fold change) between the two groups of mice plotted against the level of statistical significance. Dotted horizontal lines represent differential expression differences, and the dotted vertical line represents a significant level of *P* < 0.05. (d) GO differential gene enrichment analysis of the role of the top 30 differentially expressed proteins upregulated in biological processes.

To further disclose the biological functions of these 281 DEPs, GO enrichment was analyzed. A heatmap of the significantly altered proteins in the control and BAK groups is shown in Figure 4. Red indicates upregulation, and blue represents downregulation in protein expression (Figure 4(a)).  Figure 4(c) is a volcano plot for identifying proteins. Red spots on the right indicate significantly upregulated protein expressions, black spots on the left indicate downregulated protein expressions, and black spots indicate an absence of statistically significant differences in protein expressions. The greater the ordinate value corresponding to the point, the greater the corresponding difference in protein expression. Similarly, the greater the absolute value of the abscissa corresponding to the point, the greater the corresponding difference in protein expression. There were 281 statistically significant DEPs identified with upregulated DEPs having an abscissa value of >1.2 and downregulated DEPs having an abscissa value of <0.833. GO enrichment analysis using biological processes indicated that proteins were significantly related to the monocarboxylic acid, lipid, carboxylic acid, oxoacid, and organic acid metabolic processes (Figure 4(b)).

GO differential gene enrichment analysis of psoralen in rat liver tissue showed that TOP30 differentially expressed proteins were upregulated, of which 23 were enriched in biological processes, and the most important was metabolic processes, of which lipid metabolism ranked first and 7 were enriched in molecular functions. The results are shown in Figure 4(d).

**Figure 5:**
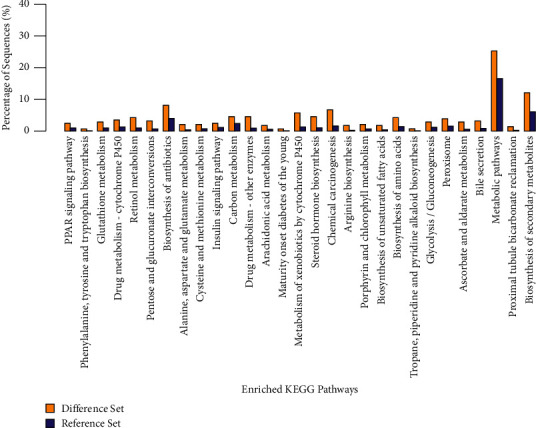
Enriched KEGG pathway (difference set: control group; reference set: BAK group).

KEGG database was used to analyze the pathways related to the toxic mechanism of differentially expressed proteins in vivo. A total of 225 pathways (at least one differentially expressed protein in each pathway) were found, which are arranged from large to small by differential protein correlation; see Figure 5. It can be seen that these differential proteins mainly affect multiple metabolic pathways. The differentially expressed proteins mainly affect chemical carcinogenesis, metabolism of cytochrome P450, drug metabolism-other enzymes, steroid hormone biosynthesis, mutual transformation of pentose and glucuronic acid in retinol metabolism, metabolic pathways, bile secretion, biosynthesis and metabolism of amino acids, and drug metabolism-cytochrome P450 and PPAR signaling pathways. Bile secretion (9 differentially expressed proteins) and glycerol ester metabolic pathway (7 differentially expressed proteins) were closely related to the biochemical and pathological results of bile in rats treated with psoralen.

### 3.5. Inhibitory Effect of BAK on the Proliferation of L02 Cells

**Figure 6:**
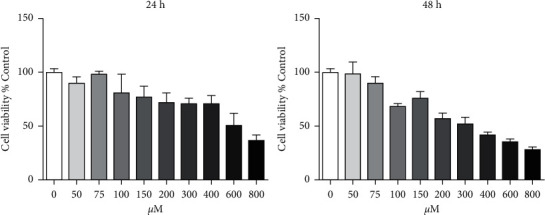
Inhibitory effect of BAK on the cell viability of L02 cells. L02 cells were incubated for 0 *μ*M–800 *μ*M BAK for 24 h and 48 h. Cell viability was measured using MTT assay. Results are mean ± SEM (*n* = 3).

MTT assay was applied to assess the effect of BAK on the IC50 of L02 cells. As shown in Figure, the IC50 of BAK treated with L02 cells for 24 hours and 48 hours was 602 *μ*M and 310 *μ*M (Figure 6). It can provide experimental conditions for investigating the changes in gene and protein expression induced by BAK in L02 cells.

### 3.6. mRNA Expression of FXR, SHP, CYP7a1, and BSEP in the In Vitro Experiments

**Figure 7:**
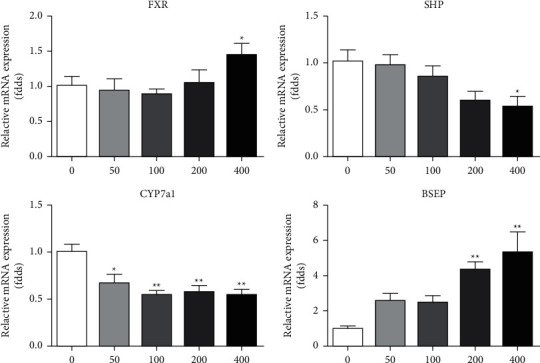
mRNA level of FXR, SHP, CYP7a1, and BSEP in L02 cells. Results are mean ± SEM (*n* = 3). ^*∗*^*P* < 0.05 and ^*∗∗*^*P* < 0.01 versus the control.

FXR plays an important role in regulating bile acid metabolism, while CYP7a1 is involved in regulating bile acid biosynthesis; SHP and BSEP are involved in regulating bile acid efflux and secretion. The effect of BAK on genes related to bile acid metabolism was detected in L02 cells. L02 cells were treated with different concentrations of BAK (50, 100, 200, and 400 *μ*M) for 24 hr. PCR results showed (Figure 7) that BAK could upregulate the expression of FXR, downregulate the expression of SHP and CYP7a1 in a dose-dependent manner, and upregulate the expression of BSEP. It is suggested that BAK may cause the disorder of bile acid metabolism in L02 cells, increase the content of bile acid in liver cells, activate FXR and BSEP, and negatively feedback inhibit the expression of SHP and CYP7a1.

### 3.7. Protein Expression Analysis Associated with BAK-Induced Bile Acid Metabolism

**Figure 8:**
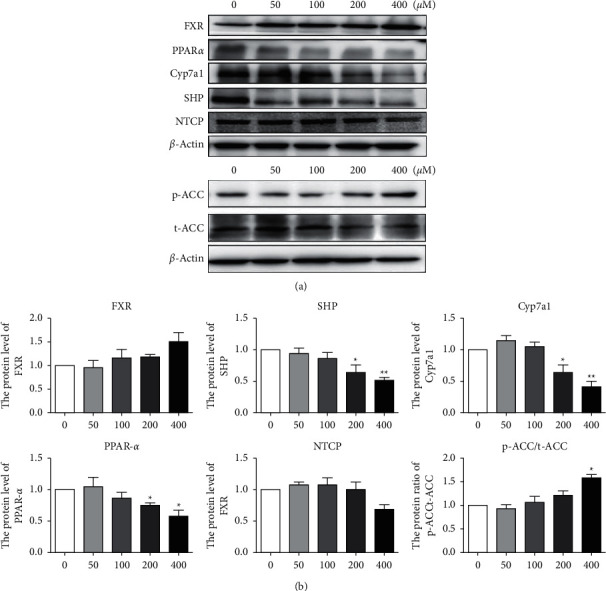
Protein levels of FXR, SHP, CYP7a1, PPAR, NTCP, and ACC in L02 cells treated with BAK (0, 50, 100, 200, and 400 *μ*M) for 48 h. Data are expressed as means ± SEM (*n* = 3). ^*∗*^*P* < 0.05^*∗*^*P* < 0.05 and ^*∗∗*^*P* < 0.01 versus the control.

To further investigate the possible effect of BAK on the expression of genes related to bile acid metabolism, we examined genes and proteins related to BAs synthesis and regulation by Western blot. The results showed (Figure 8) that BAK above 200 *μ*M could significantly reduce the protein contents of PPAR*α*, SHP, and CYP7a1, with a significant difference (*P* < 0.05). BAK above 100 *μ*M could increase the protein contents of FXR and p-ACC/t-ACC. Low concentration of BAK (400 *μ*M) could increase the expression of NTCP in LO2 cells, and high concentration of BAK (400 *μ*M) could reduce its expression.

## 4. Discussion

Studies have found that BAK has important value in skin beauty and pharmacology, but its potential hepatorenal toxicity affects its application and development. It has been found that BAK can increase serum ALT, TBIL, and TBA and reduce CYP7a1, HMG-CoA, PPAR*α*, and SREBP-2 mRNA expression, resulting in cholestasis hepatotoxicity. However, the research on its toxicological target molecule and core pathway is still ongoing, and the hepatotoxicity caused by BAK is still largely unclear. In this study, quantitative proteomics was used to directly detect the changes in protein levels, and LC-MS/MS analysis results were combined to identify the key molecules and signaling pathways of BAK-induced hepatotoxicity.

In this study, SD rats were orally given 52.5 mg/kg and 262.5 mg/kg BAK. Bile biochemical and histopathological analysis showed that BAK had hepatotoxicity. Bile biochemical results showed that the content of TBA, DBIL, and TBIL decreased, which may be related to cholestasis caused by liver cell injury or hepatotoxic injury, thereby hindering the excretion of TBA. On the other hand, the decrease in DBIL and TBIL concentrations is due to the slower liver metabolism caused by hepatotoxic injury. Histological examination showed mild hepatocyte steatosis and cholestasis in the low-dose group and hepatocyte steatosis, cholestasis, inflammatory infiltration of hepatocytes, and bile duct hyperplasia in the high-dose group, which were significantly different from those in the control group. On the other hand, the decrease of DBIL and TBIL levels in bile is related to the impairment of bilirubin uptake by hepatocytes caused by hepatotoxicity [10]. It was found that when the OATP1B1 and OATP1B3 genes were deleted, the TBIL released into the blood by MRP3 on the basal membrane of hepatocytes could not be reuptake by these two transporters, resulting in the increase of DBIL and TBIL concentrations in serum [11].

The liver is an important part of drug metabolism and detoxification. More than 80% of the liver blood flow comes from the gastrointestinal tract [12], so the liver is highly sensitive to toxicity. The DEPs identified in the liver tissue of the BAK treatment group were composed of 215 upregulated proteins and 66 downregulated proteins. The results showed that the differential proteins were enriched in bile secretion and PPAR signaling pathway in rat liver after BAK treatment, which were closely related to the results in vivo. The mechanism of bile formation, secretion, and excretion is very complex; when a variety of reasons cause bile formation, secretion and excretion disorders can lead to cholestasis [13]. FXR is a member of the nuclear receptor superfamily l H subfamily (nrlh4). Its physiological function is through the binding of the FXR/retinoid *X* receptor (RXR) dimer to the corresponding DNA response elements, thus regulating the expression of target genes [14]. FXR is a nuclear receptor that regulates gene transcription in the liver and is a bile sensor. Bile acid is the endogenous ligand of FXR, which can activate FXR to block the transcription of the CYP7a1 gene, activate the secretion and transport of bile acid, and protect the liver from damage to the liver and bile ducts caused by accumulation of toxic BAs [15]. The conversion of cholesterol to bile acid occurs in hepatocytes and is catalyzed by cytoplasm, endoplasmic reticulum, mitochondria, cell sap, and peroxidase. CYP7a1 is the only rate-limiting enzyme in the classical pathway of bile acid biosynthesis [16]. At the gene level, CYP7a1 is mainly regulated by cholesterol and bile acid, and it has been clarified that a variety of nuclear receptors are involved in the regulation of CYP7a1 expression [17]. After FXR was activated by bile acid, the related downstream effects included downregulation of bile acid synthase CYP7a1 [15], downregulation of basal uptake (NTCP) and efflux (MRP3) transporter expression, upregulation of basolateral efflux transporter (BSEP) expression, reduction of bile acid concentration in hepatocytes through negative feedback, and regulation of bile acid homeostasis [18,19]. FXR and CYP7a1 jointly form a transcriptional activation/inhibition cascade network to maintain the dynamic balance of bile acid synthesis and lipids in the body [20].

At the gene level, CYP7a1 is mainly regulated by cholesterol and bile acid, and BAK can downregulate the expression of protein and mRNA in LO2 cells. BAK can inhibit TC and TG biosynthesis [6]. When the TG level of hepatocytes decreased, the activation of liver *X* receptor *α* (LXR*α*) was inhibited, which further inhibited CYP7a1 transcription. However, iTRAQ analysis showed that the CYP7a level increased, suggesting that the body could increase the expression of CYP7a1 in a negative feedback manner when fat accumulation occurred in the liver to accelerate the transformation of cholesterol to BAs and maintain the balance of liver lipid metabolism [21]. The most important physiological function of FXR is its negative feedback on cholic acid biosynthesis. A significant increase in bile acid level can activate FXR to initiate the transcriptional activity of a series of genes, downregulate bile acid synthase CYP7a1 [22], and reduce the concentration of bile acid in hepatocytes through negative feedback. The results showed that the expression of FXR protein was upregulated, the expression of BSEP transporter was upregulated, but the expression of NTCP protein was upregulated. The upregulation of NTCP expression is considered to be compensatory, which helps hepatocytes to excrete accumulated cholic acid and reduce their toxic damage, which may be a self-protective response of hepatocytes. Chenodeoxycholic acid (CDCA) is the main component of bile acid and the endogenous ligand of FXR. CDCA combines with FXR to induce the expression of small SHP, and SHP is a transcriptional suppressor. Then, SHP interacts with transactivator LRH-1 and prevents it from activating its target genes CYP7a1 and SHP. Therefore, SHP inhibits the expression of CYP7a1 and encodes its own genes. ACC is a rate-limiting enzyme in fatty acid synthesis, which is mainly distributed in hepatocytes and adipocytes [23]. ACC gene transcription is regulated by regulatory factors such as sterol regulatory element binding protein 1 (SREBP-1) and FXR receptor. Among them, SREBP-1 is a key regulator of lipid metabolism-related enzymes, and its expression can upregulate the level of ACC, thus promoting the synthesis of fatty acids [24]. ACC can catalyze acetyl-CoA to malonyl-CoA. The presence of malonyl-CoA directly leads to the increase of fatty acid synthesis and the decrease of fatty acid oxidation and finally leads to the accumulation of fatty acids in the body. BAK can significantly reduce the level of cholesterol, thus upregulating the level of ACC and promoting the synthesis of fatty acids.

Cholestasis induced by BAK may also be related to the lipid metabolism pathway mediated by the PPAR signal pathway. The results showed that BAK could inhibit the expression of PPAR*α* mRNA and protein in LO2 cells. PPAR*α* is a ligand-activated transcription factor, which is highly expressed in the liver and can be activated by fatty acids, various lipids, and peroxidase proliferators [25]. PPAR*α* is the main regulator of liver lipid metabolism. In addition, PPAR*α* can also inhibit inflammation and acute response. The expression levels of PPAR*α* mRNA in human and mouse livers are similar, and the expression of PPAR*α* in the liver is decreased in patients with various liver injuries. PPAR*α* can effectively induce the expression of many genes involved in many lipid metabolic pathways, including microsomal, peroxisome, mitochondrial fatty acid oxidation, fatty acid binding and activation, fatty acid extension and desaturation, TG synthesis and decomposition, lipoprotein metabolism, gluconeogenesis, bile acid metabolism, and various other metabolic pathways and genes. In addition to helping to correct dyslipidemia, PPAR*α* agonists may be helpful in the treatment of cholestatic liver disease [26], nonalcoholic fatty liver disease, and type 2 diabetes. PPAR*α* can specifically bind to the peroxidase peroxisome proliferator response element (PPRE) in the promoter of the key fatty aldehyde dehydrogenase (ALDH) gene of 4-hydroxynonenal (4-HNE) metabolism [27], upregulate the expression of fatty aldehyde dehydrogenase (FALDH), and accelerate the clearance of oxidation product 4-HNE [28, 29]. The deficiency or inhibition of PPAR*α* expression can reduce the transcription level of a series of proteins and enzyme genes related to fatty acid metabolism in the liver, reduce the oxidation of fatty acids in the liver, impair the synthesis and metabolism of lipoproteins, and make fat deposition and inflammation in hepatocytes, resulting in liver injury.

It is well known that this is the first attempt to determine the molecular and signal pathways associated with cholestasis caused by BAK. These liver tissue samples were analyzed using iTRAQ technology, and the 281 DEPs detected in this study provide a solid basis for future bioinformatics analysis. In order to further verify the hypothesis that BAK causes cholestatic liver injury by affecting the PPAR pathway and bile metabolism pathway, we carried out experiments in vitro in L02 cells. The results showed that BAK had a certain degree of cytotoxicity to L02 cells in vitro. Its toxic effect was mainly to inhibit cell proliferation and did not cause obvious cell death. The results of mitochondrial staining suggested that BAK could significantly reduce the number of mitochondria. It is consistent with our study that BAK can increase the concentration of bile acid in LO2 cells and activate FXR to further block the expression of CYP7a1 gene and upregulate the expression of PPAR*α* downregulated by BESP. It leads to the disorder of the internal environment of bile acid in the liver, affects the regulation, synthesis, and transport of bile acid, and damages the synthesis and metabolism of lipoprotein through the pathway of lipid metabolism.

In summary, most of the DEPs detected in this study are rich in GO and KEGG enrichment analysis, which showed the PPAR signal pathway, bile secretion, lipid metabolism, and cytochrome P450 signal pathway. This study enables us to identify some biological processes and signal pathways related to cholestasis caused by BAK. These signals and pathways show significant changes, but these mechanisms remain to be studied.

## Figures and Tables

**Table 1:** The primers used for real-time quantitative PCR (human).

Gene			NCBI
FXR	Forward primer	5′-GAATGACCACAAGTTCACC-3′	NC_005106.4
	Reverse primer	5′-AAGAAGGGAAGTCCAATACC-3′	

SHP	Forward primer	5′-ACCTGCAACAGGAGGCTCACT-3′	NC_005104.4
	Reverse primer	5′-TGGAAGCCATGAGGAGGATTC-3′	

BSEP	Forward primer	5′-TGGAAAGGAATGGTGATGGG-3′	NC_005102.4
	Reverse primer	5′-CAGAAGGCCAGTGCATAACACA-3′	

NTCP	Forward primer	5′-GTCCAACCTCTTCACCCTGG-3′	NC_005105.4
	Reverse primer	5′-ACGATGCTGAGGTTCATGTCC-3′	

*β*-Actin	Forward primer	5′-GCGTGACATTAAGGAGAAGAATG-3′	NC_005103.4
	Reverse primer	5′-GAAGGAAGGCTGGAAGAG-3′	

## Data Availability

The data used to support the findings of this study are available from the corresponding author upon request.
